# Females with ADHD: An expert consensus statement taking a lifespan approach providing guidance for the identification and treatment of attention-deficit/ hyperactivity disorder in girls and women

**DOI:** 10.1186/s12888-020-02707-9

**Published:** 2020-08-12

**Authors:** Susan Young, Nicoletta Adamo, Bryndís Björk Ásgeirsdóttir, Polly Branney, Michelle Beckett, William Colley, Sally Cubbin, Quinton Deeley, Emad Farrag, Gisli Gudjonsson, Peter Hill, Jack Hollingdale, Ozge Kilic, Tony Lloyd, Peter Mason, Eleni Paliokosta, Sri Perecherla, Jane Sedgwick, Caroline Skirrow, Kevin Tierney, Kobus van Rensburg, Emma Woodhouse

**Affiliations:** 1 Psychology Services Limited, PO 1735, Croydon, London, CR9 7AE UK; 2 Department of Psychology, Reykjavik University, Reykjavik, Iceland; 3 Social, Genetic and Developmental Psychiatry Centre, Institute of Psychiatry, Psychology & Neuroscience, Kings College London, London, UK; 4 Service for Complex Autism and Associated Neurodevelopmental Disorders, South London and Maudsley NHS Foundation Trust, Michael Rutter Centre, London, UK; 5 Oxford ADHD and Autism Centre, Oxford, UK; 6 ADHD Action, Harrogate, North Yorkshire, UK; 7 CLC Consultancy, Perth, UK; 8 Manor Hospital, Oxford, UK; 9 National Autism Unit, Bethlem Royal Hospital, South London and Maudsley NHS Foundation Trust, Beckenham, UK; 10 Forensic and Neurodevelopmental Sciences, Institute of Psychiatry, Psychology, and Neuroscience, London, UK; 11 South London & Maudsley NHS Foundation Trust, Maudsley Health, Abu Dhabi, UAE; 12 Department of Psychology, Institute of Psychiatry, Psychology and Neuroscience, King’s College London, London, UK; 13 Independent Consultant in Child and Adolescent Psychiatry, Private Practice, London, UK; 14 Michael Rutter Centre, South London and Maudsley Hospital, London, UK; 15 Koc University, Istanbul, Turkey; 16 ADHD Foundation, Liverpool, UK; 17 ADHD and Psychiatry Services Limited, Liverpool, UK; 18 Tavistock and Portman NHS Foundation Trust, London, UK; 19 St Thomas’ Hospital London, London, UK; 20 Faculty of Nursing, Midwifery & Palliative Care, King’s College London, London, UK; 21 Cambridge Cognition, Cambridge, UK; 22 School of Psychological Science, University of Bristol, Bristol, UK; 23 Neuropsychiatry Team, National Specialist CAMHS, South London and Maudsley NHS Foundation Trust, London, UK; 24 Adult ADHD and AS Team & CYP ADHD and ASD Service in Northamptonshire, Northampton, UK; 25 Compass, London, UK

**Keywords:** Attention-deficit/hyperactivity disorder (ADHD), Female, Girls, Women, Identification, Treatment, Interventions, Comorbidity, Consensus, UKAP

## Abstract

**Background:**

There is evidence to suggest that the broad discrepancy in the ratio of males to females with diagnosed ADHD is due, at least in part, to lack of recognition and/or referral bias in females. Studies suggest that females with ADHD present with differences in their profile of symptoms, comorbidity and associated functioning compared with males. This consensus aims to provide a better understanding of females with ADHD in order to improve recognition and referral. Comprehensive assessment and appropriate treatment is hoped to enhance longer-term clinical outcomes and patient wellbeing for females with ADHD.

**Methods:**

The United Kingdom ADHD Partnership hosted a meeting of experts to discuss symptom presentation, triggers for referral, assessment, treatment and multi-agency liaison for females with ADHD across the lifespan.

**Results:**

A consensus was reached offering practical guidance to support medical and mental health practitioners working with females with ADHD. The potential challenges of working with this patient group were identified, as well as specific barriers that may hinder recognition. These included symptomatic differences, gender biases, comorbidities and the compensatory strategies that may mask or overshadow underlying symptoms of ADHD. Furthermore, we determined the broader needs of these patients and considered how multi-agency liaison may provide the support to meet them.

**Conclusions:**

This practical approach based upon expert consensus will inform effective identification, treatment and support of girls and women with ADHD. It is important to move away from the prevalent perspective that ADHD is a behavioural disorder and attend to the more subtle and/or internalised presentation that is common in females. It is essential to adopt a lifespan model of care to support the complex transitions experienced by females that occur in parallel to change in clinical presentation and social circumstances. Treatment with pharmacological and psychological interventions is expected to have a positive impact leading to increased productivity, decreased resource utilization and most importantly, improved long-term outcomes for girls and women.

## Background

Attention-deficit/hyperactivity disorder (ADHD) is a common neurodevelopmental condition described in diagnostic classification systems (ICD-10, DSM-5 [1, 2]). It is characterised by difficulties in two subdomains: inattention, and hyperactivity-impulsivity. Three primary subtypes can be identified: predominantly inattentive, hyperactive-impulsive, and combined presentations. Symptoms persist over time, pervade across situations and cause significant impairment [3].

ADHD is present in childhood and symptoms tend to decline with increasing age [4], with consistent reductions documented in hyperactive-impulsive symptoms but more mixed results regarding the decline in inattentive symptoms [5–7]. This trajectory does not appear to be different in affected males or females [6, 8]. A meta-analysis of longitudinal studies published in 2005 showed that up to one-third of childhood cases continued to meet full diagnostic criteria into their 20s, with around 65% continuing to experience impairing symptoms [9]. More recent studies in large clinical cohorts indicate that persistence of ADHD into adulthood may be much more common. Two studies from child mental health clinics in the UK and the Netherlands have reported persistence in around 80% of children with the combined type presentation into early adulthood [10, 11], potentially relating to the high severity of ADHD in this group and the use of more objective ratings [12]. The proportion meeting full diagnostic criteria for ADHD then continues to decline in adult samples [13]. Simultaneously, experiences of ADHD symptoms often change over the course of development: hyperactivity may be replaced by feelings of ‘inner restlessness’ and discomfort; inattention may manifest as difficulty completing chores or work-based activities (e.g. filling out forms, remembering appointments, meeting deadlines) [1].

Psychiatric comorbidity is very common, which may complicate identification and treatment [14]. In children with ADHD this includes conduct disorder (CD), oppositional defiant disorder (ODD), disruptive mood dysregulation disorder, autism spectrum disorder (ASD), developmental coordination disorder, tic disorders, anxiety and depressive disorders, reading disorders, and learning and language disorders [15–17]. Comorbid conditions are also extremely common in adults and include ASD, anxiety and depressive disorders, bipolar disorder, eating disorders, obsessive compulsive disorder, substance use disorders, personality disorders, and impulse control disorders [18, 19].

Prevalence of ADHD is estimated at 7.1% in children and adolescents [20], and 2.5-5% in adults [4, 21], and around 2.8% in older adults [22]. Sex differences in the prevalence of ADHD are well documented. Clinical referrals in boys typically exceed those for girls, with ratios ranging from 3-1 to 16-1 [23]. The discrepancy of ADHD rates in community samples remains significant, although it is less extreme, at around a 3-1 ratio of boys to girls [4]. Nevertheless the discrepancy in the sex-ratio between clinic and community samples highlights that a large number of girls with ADHD are likely to remain unidentified and untreated, with implications for long-term social, educational and mental health outcomes [24].

This disparity in prevalence between boys and girls may stem from a variety of potential factors. The contribution of greater genetic vulnerability, endocrine factors, psychosocial contributors, or a propensity to respond negatively to certain early life stressors in boys have been proposed or investigated as potential contributors to sexual dimorphism in prevalence and presentation [25, 26]. Whilst in childhood there is a clear male preponderance of ADHD, in adult samples sex differences in prevalence are more modest or absent [21, 27–29]. This may be due to a variety of factors, with potential contributions from the greater reliance on self-report in older samples, greater persistence in females alongside increased levels of remission in males, and potentially more common late onset cases in females [25, 26, 28].

Comprehensive views of the aetiology of ADHD incorporate biological, environmental and cultural perspectives and influences [25]. Substantial genetic influences have been identified in ADHD [30]. Individuals who have ADHD are more likely to have children, parents and/or siblings with ADHD [31, 32]. The ‘female protective effect’ theory suggests that girls and women may need to reach a higher threshold of genetic and environmental exposures for ADHD to be expressed, thereby accounting for the lower prevalence in females and the higher familial transmission rates seen in families where females are affected [33, 34]. Research suggests that siblings of affected girls have more ADHD symptoms compared with siblings of affected boys [33, 34].

There is increasing recognition that females with ADHD show a somewhat modified set of behaviours, symptoms and comorbidities when compared with males with ADHD; they are less likely to be identified and referred for assessment and thus their needs are less likely to be met. It is unknown how often a diagnosis of ADHD is being missed or misdiagnosed in females, but it has become clear that a better understanding of ADHD in girls and women is needed if we are to improve their longer-term wellbeing and functional and clinical outcomes [35, 36].

This report provides a selective review the research literature on ADHD in girls and women, and aims to provide guidance to improve identification, treatment and support for girls and women with ADHD across the lifespan, developed through a multidisciplinary consensus meeting according to the clinical expertise and knowledge among attendees. To support medical and mental health practitioners in their understanding of ADHD in females, we provide consensus guidance on the presentation of ADHD in females and triggers for referral. We establish specific advice regarding the assessment, interventions, and multi-agency liaison needs in girls and women with ADHD.

In line with previous definitions, we use the terms sex to identify a biological category (male/female), and gender to define a social role and cultural-social properties [37]. However, we acknowledge the complex differences between the sexes that occur independently of ADHD status [38], and discuss both biological differences and social roles in the current consensus.

## Methods

The consensus aimed to provide practical guidance to professionals working with girls and women with ADHD, drawing on the scientific literature and the professional experience of the authors. To achieve this aim, professionals specialising in ADHD convened in London on 30th November 2018 for a meeting hosted by the United Kingdom ADHD Partnership (UKAP; www.UKADHD.com). Meeting attendees included experts in ADHD across a range of mental health professions, including healthcare specialists (nursing; general practice; child, adolescent and adult psychiatry; clinical and forensic psychology; counselling), academic, educational and occupational specialists. Service-users and ADHD charity workers were also represented. Attendees engaged in discussions throughout the day, with the aim of reaching consensus.

The meeting commenced with presentations of preliminary data obtained from (1) an ongoing systematic review on the clinical and psychosocial presentation of females in comparison with males with ADHD (currently being led by SY and OK); and (2) epidemiological research on sex differences in self-reported ADHD symptoms in population based adolescent cohorts. Following a question and answer session, attendees then separated into three breakout groups. Each group was tasked with providing practical solutions relevant to their assigned topic. Discussions were facilitated by group leaders and summarized by note-takers. Following the small-group work, all attendees re-assembled. Group leaders then presented findings to all meeting attendees for another round of discussion and debate, until consensus was reached. Group discussions included the following themes:

1. 1: Identification and assessment of ADHD in females
  1.1 Presentation in females and what might trigger referral?
  1.2 Considering sex differences when conducting ADHD assessments
2. 2: Interventions and treatments for ADHD in females
  2.1 Pharmacological
  2.2 Non-pharmacological
3. 3: Multi-agency liaison
  3.1 Educational considerations
  3.2 Other multi-agency considerations

Taking a lifespan perspective, each theme was explored in relation to specific age groups considered to be associated with pertinent periods for environmental and biological change, and change in clinical needs and presentation. Recommendations that differed between age groups are presented separately.

The consensus group incorporated evidence from a broad range of sources. However, the assessment, pharmacological treatment, and multiagency support features reflect clinical practice and legislature in the United Kingdom (UK), and may differ in other countries.

All consensus proceedings, including group and feedback sessions were video-recorded and transcribed. One note-taker was allocated to each breakout group, and notes were subsequently circulated to each breakout group contributor for review and agreement. All materials were sent to the medical writer, who consolidated the meeting transcription, electronic slide presentations and small-group notes. The lead author worked closely with the medical writer to synthesise the consensus report, which was then circulated to all authors for review and feedback. A final draft was circulated to all authors for agreement and approval.

## Results and consensus outcome

### Presentation of ADHD in females

Although much of the scientific literature indicates an overlap in the clinical presentation of males and females with ADHD, the available evidence often draws on predominantly male samples [39] due to the higher prevalence of ADHD in males [4]. Some sex differences have been reported, which are described below, and briefly summarised in Table 1.

**Table 1:** Summary of key points for detection of ADHD in females

***ADHD symptoms:***	
• Females present with both inattentive and hyperactive-impulsive symptoms	
• Symptom severity may be lower in females than in males, particularly for hyperactive-impulsive symptoms.	
• Inattention in girls and women with ADHD may present as being easily distracted, disorganised, overwhelmed and lacking in effort or motivation.	
• Symptoms are pervasive and impairing rather than transient or fluctuating.	
• ADHD symptoms may become more obvious later in females, often during periods of social or educational transition.	
• Adult women may develop awareness of their difficulties leading them to self-present to primary services.	
• Symptoms may be exacerbated by hormonal changes during menstrual cycle, pregnancy and menopause.	
• Gender-based biases in teachers and parents appear to affect referral likelihood.	
• Less overt ADHD symptoms are less likely to lead to referral which means that inattentive girls are more often missed.	
***Comorbidity:***	
• In girls and women with ADHD common comorbidities appear more internalised in nature.	
• Whilst externalising behaviours and conditions may present in females with ADHD, these are less common than in males with ADHD.	
• Females may suffer more general impairments in intellectual functioning.	
• Risk of substance use disorders is elevated for both males and females with ADHD.	
• Internalising symptoms secondary to, or comorbid with ADHD may be misinterpreted as primary conditions. Low mood, emotional lability, or anxiety may be especially common in females with ADHD.	
• The key message is not to discount ADHD in females because they do not display the behavioural problems commonly associated with ADHD in males.	
***Associated features and vulnerabilities***	
• Difficulties with emotional lability and emotional dysregulation may be more severe or common in girls and women with ADHD.	
• Social problems may be particularly impairing.	
• Girls with ADHD are vulnerable to bullying, including physical and social-relational bullying, and cyberbullying.	
• Females with ADHD tend to become sexually active earlier than their peers and have an increased number of sexual partners. Rates of contraction of sexually transmitted infections and rates of teenage, early and unplanned pregnancies are elevated.	
• Antisocial behaviour may also be present in females with ADHD. The rate of ADHD among prisoners is similar for male and female offenders.	
• Increased school dropout, academic under-achievement.	
• Decreased self-esteem and self-concept	
• Increased rate of accidents.	
***Compensatory and coping behaviours:***	
• Compensatory behaviours may mask behaviour and impairments, and delay time to referral.	
• Dysfunctional strategies, such as drinking alcohol or smoking cannabis may be used to cope with emotional turmoil, social isolation and rejection.	
• Some girls may seek to build social support through high risk activities (e.g. joining a gang, promiscuity, criminal activities).	

#### ADHD symptoms

Research in population-based samples indicates that for both sexes the hyperactive-impulsive type predominates in pre-schoolers, whereas the inattentive-type is the most common presentation from mid-to-late childhood and into adulthood [4, 21]. By contrast, clinical studies typically report a greater prevalence of combined-type ADHD [5, 12, 22]. Early meta-analyses of gender effects have found lower severity of hyperactivity-impulsivity [40], or all ADHD symptoms (inattention, hyperactivity, impulsivity) [24] in girls than boys, although individual studies show more mixed results [8, 35, 41, 42].

Inconsistent findings may reflect that clinic referral and diagnosis tends to favour combined subtypes equally across genders, whilst community sampling points to greater prevalence of inattentive type ADHD in girls than in boys [43]. Hyperactive-impulsive symptoms have been linked to higher clinic ascertainment rates [4], and may be more commonly seen in boys [40], with inattention symptoms being less obvious and therefore less likely to be detected. These differences may lead to the perception that females with ADHD are less impaired [44].

People may experience and respond to the same behaviour of males and females in different ways due to gender-related behavioural expectations [42]. For example in two studies where teachers were presented with ADHD-like vignettes, when simply varying the child’s name and pronouns used from male to female, boys names were more likely to be referred for additional support [45] and considered more suitable for treatment [46]. Parents may also underestimate impairment and severity of hyperactive/impulsive symptoms in girls whilst over-rating these same symptoms in boys [47]. Compensatory behaviours in girls, such as socially adaptive behaviour, compliance, increased resilience [47] or coping strategies to mask behaviour [48] may also contribute to differing perceptions that may in turn prevent referral.

Less is known about the presentation of ADHD in older adults but evidence suggests whilst symptoms tend to decline, ADHD may persist into middle and old age, with a more even male-to-female community prevalence and referral rate with increasing age [22, 49].

#### Comorbidity

Externalising problems are more prevalent in males with ADHD [24], manifesting as higher rates of comorbid oppositional defiant disorder (ODD) and conduct disorder (CD) [40], characterised by rule-breaking behaviour [50, 51] and fights in school [36]. In adulthood, men with ADHD more commonly show antisocial behaviours characteristic of antisocial personality disorder [52–54]. Whilst these problems are more prevalent in males, they typically remain elevated in individuals with ADHD across both sexes in comparison with the general population. The lower rates of disruptive behavioural problems in females may contribute to lower rates of referral for ADHD assessment and support [48, 55].

Compared with males with ADHD, internalising disorders (e.g. emotional problems, anxiety, depression) are more often reported in females [24, 29, 47, 51, 53, 56]. Borderline personality traits in ADHD tend to be associated with women [57] with hyperactive/impulsive symptoms being associated with self-harming behaviours [58]. Additionally, women with ADHD have been found to be at higher risk for some adverse outcomes, including greater mental health impairment [29], severe mental illness (schizophrenia) [59] and admissions to in-patient psychiatric hospitals in adulthood [60].

The less overt presentation of ADHD in girls and women may mask the underlying condition due to females not meeting stereotypical expectations of ADHD behaviour. Instead females may be more likely to attract a primary diagnosis of internalising disorders or personality disorders, in turn delaying diagnosis and appropriate treatment [45, 47, 48].

Disordered eating behaviour has been associated with ADHD across both sexes. Whilst individual studies have shown increased disordered eating in girls and women with ADHD [53, 61], a meta-analysis of twelve studies identified increased risk of all eating disorder syndromes (bulimia nervosa, anorexia nervosa and binge eating disorder), amongst individuals with ADHD, with similar risk estimates for males and females [62]. Meta-analysis has also confirmed increased co-occurrence of obesity in children and adults with ADHD [63, 64], albeit with no difference between males and females.

Consensus meeting attendees highlighted the co-occurrence of somatic symptoms such as pain and fatigue with ADHD in females, based on clinical observation. There is little available research on sex differences in the prevalence of somatic symptoms such as pain and fatigue in people with ADHD. However, elevated ADHD symptoms have been reported in clinical cohorts with fibromyalgia [65], and chronic fatigue syndrome [66].

Young people with ADHD are at greater risk for tobacco and alcohol use in mid adolescence [67]. In adulthood they are more likely to become smokers [68], engage in higher rates of substance use [69] and develop alcohol and drug use disorders [70]. A prospective follow-up study of a nationwide birth cohort using Danish registry data reported that ADHD increased the risk of all substance use disorder (SUD) outcomes [71], with comparable risks seen for males and females. Females with ADHD (but without any comorbid conditions) had a higher risk of alcohol and cannabis abuse when compared with males.

#### Associated features, functional problems and impairments

In both children and adults ADHD is commonly accompanied by emotional lability and emotion dysregulation problems (irritability, low frustration tolerance, mood changes) [72–74]. Difficulties of this nature may be more common or severe in girls and women [30, 56–58] and emotion dysregulation problems are associated with a broad range of impairments in adulthood, including educational, occupational, social, familial, criminal, driving and financial problems [75, 76]. In an Icelandic study of ADHD symptoms in university students, poor social functioning best predicted dissatisfaction with life in males, whereas among females the best predictor of life dissatisfaction was poor emotional control [77].

Cognitive problems are well established in ADHD [78–80], spanning difficulties with executive dysfunction (such as inhibition, planning, working memory and set shifting) and non-executive cognitive domains (e.g. word reading, reaction times, colour or letter naming, response consistency). However, ADHD may also be associated with general impairments in intellectual functioning, which tends to be more prominent in females [24, 40]. Subtle social cognition deficits, including facial and vocal emotion recognition, have also been reported in both males and females with ADHD, with no clear sex-related differences [81].

A similar level of social impairment has been identified for ADHD males and females [24, 40, 82]. Autistic-like symptoms, including social and communication impairments, are common in both girls and boys with ADHD, and although these present at a higher rate in boys, likely influenced by the higher base incidence of ASD in boys, alongside greater difficulties in detecting ASD in girls [16]

Children with ADHD are more likely to experience rejection and unpopularity and have fewer friendships than their peers [83] and social problems can persist into adulthood [75]. Disruption to relationships with parents, siblings and peers has been reported for females with ADHD [84, 85]. Girls with ADHD may apply a range of ineffective strategies to resolve their peer relationship problems [86, 87], and experience more bullying than their peers [88], including physical, social-relational, and cyberbullying victimisation [23, 89, 90], whilst in boys physical victimisation appears to be more common [91]. Peer victimisation has been associated with reduced self-esteem and self-efficacy, and increased anxiety and depression symptoms in young people with ADHD [90, 91]. Adverse outcomes have been associated with interpersonal difficulties in females with ADHD including lower satisfaction with romantic relationships [92] and lower self esteem [48].

There is some evidence to suggest that elevated symptoms of ADHD are associated with excessive internet use in children and adolescents [93], as well as adults [94], but the causal direction of this association is unclear (i.e. elevated ADHD symptoms could trigger excessive internet use, or excessive internet use could lead to elevated symptoms of ADHD) [95]. Excessive gaming [96] has also been reported. It is not clear whether this association is stronger in males or females or if it is equivalent across the sexes [93, 94, 97]. A large web-based survey of adult internet behaviours and psychopathology in Norway found that elevated ADHD symptoms were associated with increased addictive technological behaviours, including social media use and gaming [98]. Overall however, addictive social media use was more common in women [98].

Throughout adolescence and the transition into adulthood, there is an increase in risk taking behaviour which may be associated with symptoms of hyperactivity and/or impulsivity [48]. For example, young people with ADHD become sexually active earlier, have more sexual partners and are more frequently treated for sexually transmitted infections [99]. Rates of teenage, early or unplanned pregnancies are elevated in girls and women with ADHD [100–102]. Pregnant women with ADHD are more likely to smoke up to the third trimester, or be obese or underweight [102].

A review of ADHD and driving reported that adults with a history of ADHD may be more likely to be unsafe or reckless drivers and have more frequent or severe crashes [103], albeit with no specific examination of sex differences. One study with data from the US National Epidemiologic Survey on Alcohol and Related Conditions, showed that reckless driving was significantly more frequent in men compared with women with ADHD, reflecting the same pattern as seen the general population [29]. This suggests that reckless driving is likely to be similarly proportionally enhanced in women as in men with ADHD.

Studies specifically reporting driving problems in women with ADHD have shown no significant association between ADHD and driving outcomes [68, 100, 104]. However, results from a prospective follow-up study of a nationwide birth cohort in Danish registers, reported increased mortality rate among individuals with ADHD; when compared with males with ADHD, females with ADHD had an increased mortality rate after controlling for comorbid CD, ODD and SUD [104]. The excess mortality in ADHD was mainly driven by deaths from unnatural causes, especially accidents. The authors speculate that the gender difference may be driven by females being less likely to be diagnosed and receive treatment than males with the disorder, leading to greater risk of accidental death.

Delinquency and criminality in females with ADHD is more common compared with their non-ADHD peers but less severe or prevalent than reported in males with ADHD [85, 105, 106]. A study examining adult criminal outcomes in children with ADHD, showed males were twice more likely to be convicted than females, but convictions in females occurred at eighteen times the rate seen in the general population [106]. Prevalence of ADHD in prison populations is estimated at 25%, with no significant differences seen in relation to gender or age [107].

### Triggers for referral

There are multiple potential triggers that may prompt the referral of females for assessment, shown in Table 2. Some of these triggers are indicative of other associated conditions and it is the clustering of multiple trait-like symptoms that are pervasive and impairing that is informative, rather than state-like symptoms showing situational change. The decision to refer would also be strongly supported if there is a first-degree relative with ADHD.

**Table 2:**
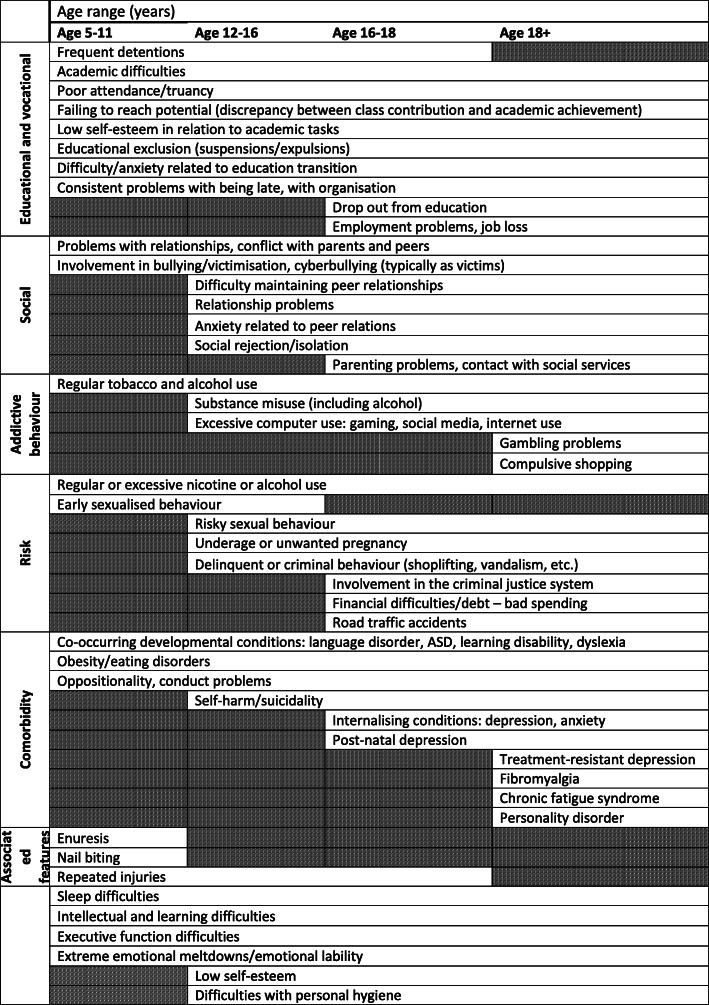
Co-occurring functional problems, features or conditions commonly seen in girls and women with ADHD

Legend: Co-occurring functional problems, associated features or conditions commonly seen in addition to ADHD symptoms in girls and women with ADHD, presented along with age-ranges for detection. These may serve as triggers to help to identify individuals who may require assessment for ADHD

The stereotype of the ADHD ‘disruptive boy’ [47] is likely to influence the likelihood of referral and access to diagnosis and treatment. The key message is not to disregard females because they do not present with the externalising behavioural problems, or the disruptive, hard-to-manage presentation (e.g. engaging in boisterous, loud behaviours) commonly associated with males with ADHD. Females with ADHD may be overlooked and/or their symptoms misinterpreted, particularly for those in highly structured environments, receiving a high level of support, and for those who have developed strategies to mask or compensate for their difficulties.

It is important to be mindful that environmental demands (including educational, occupational, financial, familial and social functions and responsibilities) increase in number, scope and complexity with age and level of independence, whilst support resources decline [108]. Many young peoples’ struggles and impairments become apparent as they lose the family and educational scaffolding that was previously in place. Therefore, young people (both males and females) may be particularly vulnerable at times of transition, when symptoms become exposed. Increased functional demands on transition to secondary school (planning ahead, organising work and juggling assignments) may lead them to feel overwhelmed. This may impact on self-esteem and result in learner anxiety and perfectionism in an attempt to compensate. Periods of transition may therefore unmask unidentified ADHD by exposing or exacerbating symptoms, together with the development of internalising problems leading to increased vulnerability.

These environmental changes often occur at a time when girls undergo changes in their physiological and sexual maturation. There is growing recognition that puberty is a phase of high risk for mental health problems [109]. The developmental changes that occur during puberty and later in adolescence may lead females with ADHD to be particularly psychologically vulnerable if they are not able to access support.

Difficulty coping with more complex social interactions and resolving interpersonal conflict may also trigger cause for concern. As girls with ADHD move into their teenage years, difficulty maintaining friendships often becomes more marked and they may feel rejected and socially isolated. Some respond with bravado to buffer them from social isolation but a ‘brave face’ is unlikely to prevent them from feeling distressed and developing low mood and anxiety. Dysfunctional coping strategies and the lack of a support network may lead them to express these feelings by self-harming behaviours (e.g. cutting) or changes in eating patterns.

The identification of specific educational or learning problems may also be an important trigger for referral. Children may be diagnosed with specific learning difficulties, such as dyslexia, when a diagnosis of ADHD may be more appropriate. Parents/carers and teachers may note the disparity between educational performance (day-to-day classroom contribution) and achievement (end grades).

Many young people with ADHD do not exceed the mandatory minimum level of schooling, and the problems described above may become even more marked when they enter further education and/or leave home. Research suggests that adolescent school girls with elevated ADHD symptoms make significantly fewer plans for their future than their peers, suggesting that they leave this to chance and opportunistic encounters [86]. Those who enter the world of work may find that their difficulties evolve into employment impairments and limitations. However, as they mature young people may begin to develop greater awareness of their difficulties, leading to an increase in self-referrals.

### Assessment

For both males and females, a comprehensive assessment should be completed to accurately capture the symptoms of ADHD across multiple settings, their persistence over time and associated functional impairments. High rates of comorbidity are typically present. The assessment process is typically tripartite involving the use of rating scales, a clinical interview and ideally objective information from informants or school reports. Key recommendations for enhancing diagnostic assessment in girls and women are provided in Table 3.

**Table 3:** Enhancing ADHD diagnostic assessment in females: consensus recommendations

Rating scales, a clinical interview and an observational assessment.	
***Rating scales***	
• Norms from predominantly male or mixed-sex samples may disadvantage female patients. Rating scales providing female norms (see Table 4) may provide cut-offs more sensitive to female presentation.	
• Where female norms are not available, greater emphasis on collateral information is required (e.g. parental and school reports).	
• Findings should be interpreted cautiously. Rigid adherence to cut-offs may lead to a high proportion of false positives and negatives.	
***Clinical interview***	
• Assessors should bear in mind that family members may also have ADHD which may affect their judgment of ‘typical’ behaviour.	
• Small modifications to symptoms may help to capture more female-centric behaviour (see topic for examples).	
• Assessors should examine factors that may mask or moderate behaviour in different settings, e.g. compensatory strategies or accommodations at home or school (both functional and dysfunctional).	
• Age-appropriate, common co-occurring conditions in females with ADHD should be explored, including ASD, tics, mood disorders, anxiety, eating disorders, fibromyalgia and chronic fatigue syndrome.	
• A risk assessment and consideration of future challenges (e.g. personal, clinical, educational, social-relational and psychosexual) is required.	
***Collateral information***	
• School reports may comment more on attentional problems (daydreaming, distracted, disorganised, lacking in motivation and effort) or interpersonal relationship problems in girls with ADHD.	
• Objective neuropsychological test results are not specific markers of ADHD but may provide useful supplementary clinical information. The QB scales have female-specific normative data and may therefore be more sensitive.	

#### Rating Scales

Rating scales can obtain perspectives from different informants (e.g. family, teacher, youth worker, occupational health practitioner) in a consistent, quick and easy way. They are not the sole domain of healthcare practitioners and can be applied (with patient consent) by allied professionals, such as social care providers and those working in educational and occupational establishments, to guide whether referral might be merited.

While rating scales are useful aids for clinical assessment and treatment monitoring, findings should be interpreted cautiously if they are used for screening purposes as they are non-specific markers of potential problems [110]. Rigid adherence to cut-offs may lead to a high proportion of false positives and negatives. There are many rating scales available with varying merits and limitations and some are yet to be updated to reflect revisions to diagnostic criteria. Where possible both informant- and patient-rated scales should be obtained. Rating scales in common use are presented in Table 4.

**Table 4:** Clinical assessment resources which are in common use for ADHD

**Rating scales**				
**Assessment name [reference ID]**	**Age range (years)**	**Application**	**Female norms**	**Free access**
Conners’ Comprehensive Behavior Rating Scales (CBRS) [111]	6-18	• Patient-rated (age 8-18) • Parent rated • Teacher rated	Yes	No
SNAP-IV R Rating Scale [112]	8-18	• Parent rated • Teacher rated	No	Yes
RATE-C [113]	8-11	• Patient-rated • Informant rated	No	Yes
Kiddie-SADS DSM-5 Screen Interview (K-SADS-PL) [114, 115]	6-18	• Patient rated • Parent rated	No	Yes
Strengths and Difficulties Questionnaire (SDQ) [116]	3-16	• Patient-rated (age 11-16) • Parent rated • Teacher rated	Yes	Yes
The Vanderbilt ADHD rating Scales (VARS) [117]	6-12	• Parent rated • Teacher rated	No	Yes
The Development and Well-being Assessment (DAWBA) [118]	2-17	• Teacher rated	No	No
RATE [113]	16-54	• Patient-rated • Informant rated	No	Yes
Conners’ Adult Rating Scales (CAARS) [119]	18+	• Patient-rated Informant rated	Yes	No
Adult ADHD Self-report Rating Scale (ASRS) [120]	18+	• Patient-rated	No	Yes
**Clinical interviews**				
**Assessment name [reference ID]**	**Age range (years)**	**Application**		**Free access**
ADHD Child Evaluation (ACE) [121]	5-16	Administered to informant (parent, carer, family member) close to patient. Patient also typically invited to contribute.		Yes
The Development and Well-being Assessment (DAWBA) [118]	5-17	Administered separately to patient (if age 11-17 years) and parent/carer		No
Young DIVA-5 [122]	5-17	Administered to patient in the presence of a parent, carer or family member (where possible)		No
ACE+ [123]	16+	Ideally administered to patient in the presence of an informant		Yes
Conners’ Adult ADHD Diagnostic Interview for DSM-IV^TM^ [124]	18+	Administered to patient		No
Diagnostic Interview of Adult ADHD (DIVA-5) [125]	Limits not specified	Administered to patient		No
Diagnostic Interview for ADHD in Adults with Intellectual Disability (DIVA-5 ID) [125]	Limits not specified	Administered to patient with intellectual disability in the presence of an informant/carer (where possible)		No

Rating scale norms are predominantly from male or mixed samples, which may disadvantage their use in females, although some provide female-specific norms (see Table 4). Where female norms are not available, greater emphasis should be placed on collateral information (e.g. parental and school reports). The Nadeau and Quinn checklists may also be used as indication of possible ADHD in girls and women [126, 127], providing structured self-enquiry of ADHD symptoms and associated problems, including a range of difficulties such as learning problems, social/interpersonal and behavioural problems.

Since hyperactive and impulsive behaviours tend to decline as patients move into adulthood and impairments associated with inattention are often sustained, it is helpful to re-administer age appropriate scales as young people with ADHD become adults.

#### The clinical interview

A clinical diagnostic interview, supplemented by a mental state examination, should consider the extent to which the individual’s functioning is age appropriate and obtain examples of how difficulties interfere with functioning and development in home and education/work environments. For children this is usually carried out in the presence of a person close to the child, has known the child for a long time, and is familiar with their developmental history and functioning in different settings (commonly a parent or carer).

Age-appropriate, common co-occurring conditions in females with ADHD should be explored, including ASD, tics, mood disorders, anxiety, and eating disorders. Fibromyalgia, chronic fatigue syndrome, body dysmorphic disorder and gender dysphoria may also be explored as possible co-occurring conditions. The assessor needs to consider what is primary (i.e. occurring alongside and independently to ADHD) and what is secondary (i.e. caused or exacerbated by ADHD). It will help to determine whether the presenting problem is trait-like or episodic in nature. Clinicians should be alert to signs of self-harming behaviours (especially cutting), which typically peak in adolescence and early adulthood [128, 129]. Substance and alcohol use disorders should also be assessed in teenagers and adults. Sleep problems are commonly seen in both males and females with ADHD [130, 131], and it is important to determine whether this primarily relates to symptoms of ADHD or co-occurring anxiety.

Since heritability of ADHD is high, ranging between 70-80% in both children and adults [132], it is important to be mindful that informants who are family members may also have ADHD (possibly undiagnosed) which may affect their judgment of ‘typical’ behaviour. The assessor should therefore obtain specific examples of behaviour from the informant and use these to make clinically informed judgments, rather than relying upon the informants’ perception of what is typical or atypical.

Semi-structured clinical diagnostic interviews are helpful as they guide the healthcare practitioner to complete a comprehensive developmental and clinical interview, whilst allowing for individual differences to be considered. For example, symptoms relating to excessive talking, blurting out answers, fidgeting, interrupting and/or intruding on others have been reported as more frequently endorsed by women than men with ADHD [53, 55] and may be more sensitive to the presentation in females. Small modifications may help to capture more female-centric behaviour (e.g. ‘excessive talking and giggling’ instead of ‘excessive talking’) [133]. Commonly used diagnostic interviews are presented in Table 4. There are three clinical interviews that prompt the assessor to consider the presence of co-existing conditions (which may differ between males and females); ACE, ACE+ [134] and the DAWBA [118].

When assessing adults, the clinical interview is usually completed with the affected individual but whenever possible collateral information should also be obtained. This may be from a parent or carer or another close member of the family. If a reliable informant cannot be identified who knew (and can recall) the individual well during their childhood, it may be helpful to obtain information from an informant who currently knows the individual well (e.g. a partner or a close friend who has known them for a significant period time, 5 years or more) in order to supplement self-reported information with a secondary perspective. If available, reports from childhood (for example, school, social service and/or previous clinical reports) are likely to be informative. Importantly, however, it may not be possible to rely on school reports when assessing females, as subtle hyperactive-impulsive symptoms may have been missed by teachers and/or they omit to comment on interpersonal or relationship problems. School reports may comment more on attentional problems (such as daydreaming or lacking in motivation and effort).

Some girls and women with ADHD become competent at camouflaging their struggles with compensatory strategies, which may lead to an underestimation of their underlying problems. Often these strategies have an adaptive or functional purpose, for example, enabling them to remain focused or sustain attention, or to disguise stress and distress. However, not all strategies are helpful. Coping strategies may be less overt, such as avoiding specific events, settings or people, not facing up to problems, spending too much time online or not seeking out help when needed. Teenage and adult females with ADHD may turn to alcohol, cannabis and other substances to manage emotional turmoil, social isolation and rejection. Some may seek to obtain a social network by forming damaging relationships (for example, joining a gang, engaging in promiscuous and unsafe sexual practices, or criminal activities). If there is cause for concern, a risk assessment should be included that enquires into suicidal ideation, the use of illicit drugs, substances and alcohol, antisocial attitudes and behaviours, harm to self and others, bullying and assault, excessive internet use, unsafe sexual practices and exploitation of a sexual, financial or social nature. In some cases, a physical health assessment may be warranted.

With older age and persistent inattentive symptoms, there may be an increasing risk that individuals with ADHD are incorrectly diagnosed with mild cognitive impairment. Self- perceived ADHD symptoms, and in particular inattention, are found to increase with age in diagnosed adults and perceived symptom severity appears to be exacerbated by concurrent depressive symptoms [49]. It is not uncommon that adults with ADHD are treated for anxiety and/or depression in the first instance. Clinicians should be mindful that those with treatment resistant anxiety and/or depression should be screened for possible undiagnosed ADHD. Indeed, careful examination of developmental history will elucidate whether symptoms are longstanding and have been exacerbated by situational or biological changes, or whether they represent new-onset symptoms that are less indicative of ADHD.

#### Objective assessments

Whenever possible, the assessor should obtain collateral information from independent sources. This may include direct observations in a specific setting (e.g. in clinic, at home or at school). A wealth of useful information may be obtained from observing a child in school and speaking directly with teachers. When assessing adults, perusal of school, college and/or employment reports (if available) can be helpful.

Tests that assess executive dysfunction may help to determine deficits in higher order processing skills such as task switching, perseveration, planning, sequencing and organising information. Some have been specifically developed for ADHD populations and focus on assessing attention, impulsivity and vigilance in children and adults. Neuropsychological tests such as the Test of Everyday Attention (TEA) / Test of Everyday Attention for Children (TEACh), may be helpful supplements to the diagnostic process. Those most commonly used in clinical practice include the Conners’ Continuous Performance Test, third edition (CPT 3 [age 8+]) [135] and the QbTest [136], the latter including a measure of hyperactivity. QbTest scales have normative data specific to each sex (age 6-60) and may therefore be more sensitive to ADHD in females. The assessor should be mindful that an individual with ADHD may perform relatively well on novel tasks, especially those presented as computerised games providing immediate gratification via rapid feedback. Moreover, findings may lack ecological validity and not reflect performance in the ‘real world’. Neuropsychological assessments are not specific markers of ADHD and should only be used to augment clinical decision making and not be used as stand-alone diagnostic tools.

### Interventions and Treatments

Prompt identification and treatment of ADHD is recommended, as there is evidence of long-term functional benefits associated with treatment [137, 138]. ADHD is typically treated with psychoactive medication, psychoeducation and therapeutic interventions at all ages, and a stronger treatment effect has been reported with multi-modal treatment [138]. A brief summary of treatment recommendations is presented in Table 5.

**Table 5:** Treating ADHD in girls and women: key consensus recommendations

***Pharmacological treatment***	
• Medication recommendations do not differ by sex and differ only modestly by age.	
• Treatment monitoring may require deviation from conventional outcomes from rating scales and behaviour management. Individualised targets (e.g. emotional lability, academic attainment) may be more appropriate.	
• Prescribing needs to consider interactions between ADHD and other medications for comorbid conditions, where applicable.	
• Where mood problems are apparent but not pervasive it is advisable to treat ADHD symptoms and monitor for improvement first, prior to considering or initiating treatment for mood disorders.	
• Appetite suppression as a side effect of stimulant medication should be considered if eating disorders are a concern.	
• Risks of substance use whilst on ADHD medications should be considered and discussed with patients.	
• Treatment with ADHD medications is generally not advised during pregnancy or breastfeeding.	
• Review is advised during and after key periods of hormonal change (menopause, pregnancy).	
• Psychoeducation on pharmacological treatment options and treatment targets for parents and affected girls may help to improve adherence and engagement.	
• Regular review is required throughout development and may be especially important at times of key transitions.	
***Non-pharmacological treatment***	
• Whenever possible, provide psychoeducation taking a lifespan approach.	
• Parents and carers of teenage girls need psychoeducation to support detection of deliberate self-harming or risky behaviour.	
• Follow-up sessions are essential for support at key points of transition.	
• Interventions should be tailored to needs and address difficulties and challenges faced at home, school/work and in social activities.	
• Both group and individual assessments may be beneficial.	
• Direct parental input into interventions is required for children. Adolescents and adults are more likely to receive direct interventions without parental/carer input.	
• Programmes for all ages will benefit from focus on ADHD symptoms and associated problems, including executive functions, emotion regulation, conduct and social impairments, in an age-sensitive manner.	
• Programmes should differ depending on age with issues relating to transition,	
• As relevant, risk (sexual risk, substance misuse), and self-management should be addressed in adolescence, with adult interventions including employment problems, child-rearing and parenting.	

In the context of changes in the presentation of ADHD with development and ageing, regular treatment reviews are advised. These can revisit and optimise current pharmacological and non-pharmacological approaches, or revisit those patients who previously may not have been suitable for specific treatments or who did not show good response.

#### Pharmacological management

ADHD is commonly treated with psychostimulants, such as methylphenidate and amphetamine. In certain cases, a nonstimulant such as atomoxetine, an extended-release form of guanfacine or clonidine, or bupropion may be prescribed, especially when stimulants are inappropriate or have been unsuccessful. These medications, with the exception of bupropion are recommended by the National Institute of Health and Care Excellence (NICE) guidance [139]. A systematic review and network meta-analysis recommended methylphenidate for children and adolescents and amphetamines for adults, taking into account both efficacy and safety [140]. Larger confidence intervals in relation to the tolerability and efficacy of bupropion, clonidine and guanfacine were reported, indicating less conclusive results with regards to the efficacy and tolerability of these oral medications [140].

Treatment recommendations do not differ by sex and differ only modestly by age (NICE, 2018 [139]). The overarching opinion in the consensus group was that there are no differences in the medicines used to treat ADHD in girls and boys. Stimulant medications show good efficacy for improving ADHD symptoms in both children [141] and adults [142], and response appears comparable in females and males [143, 144]. However girls with ADHD tend to be less likely to be prescribed stimulant treatment than boys with ADHD, and are likely to start treatment at an older age [145].

The potential benefits of treatment must be viewed in the context of lifetime adverse outcomes associated with poorly managed ADHD described previously. Prompt identification and treatment may help to improve longer-term functional, health and mental health outcomes. Reduced rates of comorbidity (including depression, anxiety disorders, and disruptive behaviour disorders) have been noted in stimulant treated ADHD populations [146, 147], although the converse effect has also been reported [148]. Comorbid ADHD is associated with treatment resistant depression [149] and regular treatment for ADHD may reduce rates of treatment resistance [150]. Pharmacological treatment of ADHD is also associated with improved educational [146] and occupational [151] outcomes, as well reduced rates of criminality [152]. Pharmacotherapy for ADHD appears to be a protective factor for obesity [64], and some limited evidence suggests that it may increase efficacy of weight management strategies (reviewed in [153]). Additionally, there appears to be a benefit of ADHD treatment with regards to substance use disorders. A study of commercial healthcare claims showed reduced emergency department visits related to substance use disorders when patients were prescribed treatment for ADHD [154].

Whilst pharmacological treatments themselves should not differ by sex, the way in which they are managed and monitored should occur in a sex-sensitive manner. The consensus group observed that prescribers need to consider ADHD presentations and associated problems in females to appropriately target what medication aims to improve. It may be less helpful to strictly adhere to conventional rating scales or focus on behaviour management to identify treatment-related changes. Instead, treatment response may be better captured through individualised targets, such as measures of emotional regulation, participation in education, and academic attainment. In the UK, all government funded schools have attainment ratings for each child, which could be examined by the prescriber prior to commencement of medications and monitored over time in conjunction with prescribing. Girls with emotional regulation difficulties (for whom internalising difficulties are often a key component of their ADHD) could benefit from measuring changes in emotional lability with medication use.

Parents and carers may not be as aware of the benefits of medication in girls, especially those with inattentive presentations in the absence of challenging or disruptive behaviour. Psychoeducation regarding available treatments and what they are targeting, provided for parents and girls with ADHD themselves, may help to ensure engagement in treatment and improve adherence to treatment regimens. Where required, adherence may be improved by using long-acting stimulant medication in place of short-acting medications [155–157].

In early to late adolescence, recommended treatment regimens in ADHD remain the same as in early childhood, and do not differ between girls and boys. The use of medication should be followed up over time to verify if medications are effective and well tolerated, and to manage the effects of related conditions (e.g. anxiety, depression) if they emerge. Side effects of stimulants need to be considered, particularly the side effect of appetite suppression if eating disorders are a concern [158].

﻿There is some early evidence to suggest that ADHD medications may differentially affect women depending on progression of their menstrual cycle. Two small studies have shown that hormonal changes during the menstrual cycle (oestrogen and progesterone levels) may impact on the subjective euphoric and stimulating effects of d-amphetamine in healthy women who are not affected by ADHD [159, 160]. Changes in subjective ratings of stimulation have also been noted in young women unaffected by ADHD in response to d-amphetamine after application of estradiol patches (commonly used to treat problems associated with menopause) [161]. Cellular and small neuroimaging studies which show early evidence of a link between dopamine systems (implicated in the aetiology of ADHD) and gonadal hormones (reviewed in 49). In a case study, a woman with ADHD showed positive response to treatment adjustment around the menstrual cycle, which included augmentation with an antidepressant (fluoxetine) during the immediate pre-menstrual period to reduce problems with moodiness, irritability and inattention normally well controlled through stimulant medication alone [162].

Whilst the evidence above does not support treatment adjustment according to the menstrual cycle, anecdotal clinical accounts were given during the consensus meeting supporting that this approach benefits certain patients. The consensus group noted that this type of regular medication adjustment may be easier to manage for adult women who can take more control of their dosing, rather than adolescent girls who tend to respond better to routine. There were also anecdotal accounts of symptom exacerbation in women during the post-menopausal period. During this time physicians may consider the use of hormone replacement therapy, if deemed beneficial.

As hormonal changes take place during puberty, the postpartum period and the menopause, patients may report changes in their symptoms and re-evaluation of treatment regimens may be helpful. It may be advised that women track their symptoms during these periods to establish patterns which may help support changes to the medication regimen when reviewed by their physician.

There is no evidence to indicate that females in either early, middle or later adulthood should be treated any differently with respect to specific medicines for ADHD symptoms. However, given the complex clinical picture of many adults with ADHD, particularly with regards to the presence of comorbid conditions, prescribers need to be mindful of potential interactions with other drugs. If ADHD treatment improves co-morbid conditions, medication regimens could potentially be simplified.

Women with ADHD are highly likely to suffer from mental illness and SUDs. Clinicians need to be mindful of, and discuss with their patients, the risks around alcohol and drug use whilst on ADHD medications. Affective symptoms (most commonly emotional lability or volatility) associated with ADHD, may be misattributed to depressive disorders. For women with ADHD in whom depressive mood symptoms are apparent but not pervasive, it is advisable to treat the ADHD symptoms first and monitor for improvement. A more consistent low mood may be due to demoralization driven by ADHD and its functional impairments, and may improve with ADHD medication.

Symptoms or problems experienced by women with ADHD may also overlap with those indicating a personality disorder, such as BPD. Careful consideration is required to establish the underlying condition(s). This will have follow-on implications for treatments, which differ significantly between personality disorders and ADHD. Biosocial theory suggests that BPD may arise as a function of the interaction of early vulnerabilities (impulsivity and heightened emotional sensitivity) with the environment [163]. If ADHD symptomatology may predispose individuals to later personality disorders [164], it is possible that early detection and appropriate treatment could prevent the later development of these conditions [165]. However, there is no clear empirical evidence supporting this hypothesis at present [109].

Historically, prescribing ADHD medication during pregnancy or breastfeeding was not advised due to a lack of evidence for safety and risks of unknown adverse effects to the baby. However, a recently published systematic review and meta-analysis reported that exposure to ADHD medication during pregnancy does not appear to be associated with serious adverse maternal or neonatal outcomes [166]. Nevertheless, the group were cautious regarding this outcome and considered that until these findings have been robustly replicated, prescribing ADHD medication during pregnancy or breastfeeding should be avoided. There may be situations however where risks of not treating ADHD may outweigh potential risks to the foetus and continued prescribing may be necessary subject to more careful obstetric monitoring. In this case, women with ADHD need to be informed of these risks.

Women may find their ADHD symptoms worsen or become particularly difficult to manage while breastfeeding given additional life pressures that occur in the presence of a new baby. Whilst it may be possible to use short acting stimulant medication, timed around breastfeeding to minimise transfer between mother and child [167], there is minimal scientific evidence to support this approach, and it would be generally safer to advise the cessation of medications during this period altogether. Where ADHD medication is necessary, then an alternative to breastfeeding is needed to minimise any risk to the baby.

Prescribers should be aware that mothers with ADHD may experience difficulties in managing their own symptoms alongside the increased demands from family life, and these difficulties may be augmented by the presence of ADHD in their own children. They may benefit from more frequent evaluations of ancillary support requirements and/or a careful review of medication dosage.

#### Non-pharmacological management

A number of meta-analyses of data from child and adolescent samples have shown that non-pharmacological interventions targeting cognitive processes show small to moderate effects on ADHD symptom outcomes when rated by individuals who are close to the treatment setting (often parents), but that effects become attenuated or non-significant when outcomes are obtained from individuals who are blinded to the interventions (often teachers) or adequately controlled active or sham conditions [168–170]. Research has documented this effect for specific interventions such as cognitive training (for example, training of attention, memory, inhibitory functions) [169], and neurofeedback [170] - although more recent research suggests that effects of neurofeedback are more modest rather than absent when assessed by probably blinded evaluators [171].

Meta-analyses also show potentially more promising outcomes from non-pharmacological interventions that target behaviours and outcomes beyond ADHD symptoms alone in children and adolescents, with ADHD intervention in children producing a moderate effect on parent stress [172], and organisational skills interventions which resulted improved ratings from both parents and teachers and with modest improvement in academic function [173]. Behavioural interventions were found to have a moderate positive effects on a range of outcomes including changes in parenting and conduct problems, even when rated by blinded assessors [174].

Meta analyses also indicate more promising results from cognitive behavioural therapy, and mindfulness interventions on ADHD symptoms in studies with primarily adult samples, albeit without comparisons from blinded raters [175, 176]. Benefits of non-pharmacological treatments in adults are also shown to range beyond improvements in ADHD symptoms, as shown in a recent report from a psychological intervention programme in adults with high levels of ADHD symptoms across three municipalities in Denmark. Participant outcomes were compared with matched controls receiving ‘treatment as usual’ drawn from the Danish Registers at 6 and 12 months post-treatment follow-up. The study showed that participation in the programme was associated with increased employment, education rates and reduced use of cash benefits and social services [177]

The efficacy of a psychological approach varies across the lifespan and the content of treatment should be tailored to meet the individual presentations and needs of individuals with ADHD [178]. Regular review of how a person is coping may be especially important at times of key transitions. Since the needs of females with ADHD differ considerably as they mature, the goals of treatment are presented across three age ranges: primary age (5-11 years), secondary age (12-18 years) and adulthood (age 18+).

##### Primary age

ADHD often places a significant psychological, emotional, and economic burden on families as well as the individual; increased stress and discord in the family unit has been reported [179, 180]. Where ADHD affects females, it is also more common in their family members [33, 34], resulting in bidirectional effects of ADHD in the mother-child relationship. The aim of non-pharmacological interventions therefore is to support individuals with ADHD and their families to develop and/or improve skills and coping strategies. Psychoeducation and psychological interventions directed at both patient and family are needed to achieve this, as they provide the tools to make helpful changes and achieve positive immediate and long-term functional outcomes.

There are two types of parenting intervention that may be offered to parents/carers in this age-group: (1) parent/carer support interventions, where people can meet and share experiences with others, and (2) parent/carer mediated interventions, sometimes referred to as ‘parent training’. The latter is an indirect intervention as the parent/carer is taught to deliver interventions to their child. Ideally both approaches should integrate a psychoeducational component as this is likely to lead to better outcomes.

Psychoeducation and interventions for girls in this age group should include discussion about the difficulties and challenges they will face at home, in school and in social activities - and how they may respond. At school this may relate to difficulty with sustaining attention, organisation, time management, planning activities, prioritising and organising tasks. They may also require generic skills for coping with interpersonal difficulties and/or social events, conflict management, emotional lability, anxiety and feelings of distress. Some girls may need interventions to address discrete problems, including sleep problems [131], enuresis [181], bullying [89, 90] and repetitive behaviours such as nail biting [182]. It is important to emphasise that problems may be less overt in females with ADHD compared with boys due to them being less boisterous and hyperactive, yet their struggles with impulse control may manifest in a different way such as blurting out hurtful things to friends and family in anger, or deliberately self-harming behaviours.

Both group and individual sessions working directly with the child may be helpful additions to parent/carer mediated treatments, although individual treatments may be more appropriate for those with severe symptoms, intellectual limitations and/or those who are unable to tolerate group sessions (e.g. due to lack of confidence, poor social communication). Two specific programmes have been developed for young children with cognitive, emotional, social and/or behavioural problems; one for individual delivery [183] and the other for group delivery [184, 185].

##### Secondary age

As children mature, they are more likely to receive direct interventions without input from their parents or carers. The best mode of psychological treatment is cognitive behavioural therapy (CBT) together with psychoeducation (which can be provided to both patients and parent/carers together or independently). Parents and carers need to be aware of the elevated risk of deliberate self-harming behaviour (e.g. cutting), eating disorders, substance abuse, risk-taking behaviours, and vulnerability to exploitation in teenage girls with ADHD. Thus psychoeducation should include indicators that problems of this nature may be developing.

The focus of treatment in this age group should include information and guidance on the need for adherence to medication. There is evidence that adherence to pharmacotherapy declines in the teenage years, although adherence appears to be modestly better in girls than in boys [155, 157, 186]. These changes have been attributed to adverse effects, sub-optimal response, reduction in parent supervision, increased need for autonomy, and social stigma associated with ADHD diagnosis and taking medication [155, 156]. It is important to provide psychoeducation to encourage young people with ADHD to understand and take ownership of their diagnosis and treatment, rather than feeling it has been imposed on them. Those diagnosed with ADHD for the first time in their teenage years are likely to require different intervention strategies to those who have been treated pharmacologically earlier in childhood. For example, psychoeducation should include information on the purposes and benefits of particular medications, as well as strategies around self-management.

Problems presenting in younger childhood often become more marked with age due to increasing academic and social expectations. These are important years in terms of a young person’s education and interventions can help to support executive function (e.g. improving skills to address problems with time management, focus, sustaining attention, organisation and planning) which may in turn support their coping in secondary schooling. Teenage girls may particularly benefit from treatment aimed at improving self-concept and identity. This may be achieved by unpacking the association between ADHD, lack of achievement, poor self-efficacy, lack of self-confidence, poor self-image and low self-esteem.

Aside from addressing core ADHD symptoms and executive deficits, specific interventions should focus on developing skills and coping strategies for co-occurring conditions, such as managing poor emotional regulation, low mood and anxiety, controlling the impulse to deliberately self-harm (including skin picking and cutting), eating for pleasure or restricting food. Additional support for new skills required in teenage years, such as managing money, may also be helpful.

In adolescence, young people develop a strong focus on peer relationships and a tendency towards social conformity [187]. For teenage girls with ADHD, the desire to develop robust and supportive social networks can be strong, and the rejection and social isolation experienced by many may mean that family support is especially valued [87]. Simultaneously interpersonal conflict with family members is not uncommon, and girls may engage with dysfunctional social groups and activities in an attempt to gain a sense of ‘belonging’ and to be accepted. Girls with ADHD are at increased risk of being victims of bullying [23, 90], and social media may provide additional challenges since it offers a public platform for victimisation.

Behavioural and oppositional problems remain elevated in teenage girls with ADHD in comparison with their peers, albeit not as elevated as in boys with ADHD. Girls with ADHD may attract detentions, suspensions or exclusions from school for their conduct or oppositional behaviour. Their behaviours may be more socially motivated (e.g. spiteful, manipulative, threatening behaviours and/or lashing out at peers) rather than overt aggression. Social skills and interpersonal relationship interventions become salient at this age. These may aim to develop coping strategies to regulate emotions, build confidence, raise self-esteem and manage peer pressure, deal with rejection and manage conflict.

Interventions to address impulsivity and associated risk-taking behaviour may be helpful. These problems may manifest in early onset of sexual behaviour. The desire to be accepted into a peer network may be a motivating factor. Girls with ADHD are more likely to be pressurised into sex or engage in risky sexual behaviour. They are also more vulnerable to sexual exploitation or perceived exhibitionism (including internet grooming, ‘sexting’ and posting inappropriate content [188]). This may result in disproportionate social stigma for adolescents and young women with ADHD, in the face of violations of social expectations of female sexuality (where promiscuity may enhance male but damage female reputations). As girls become sexually active, the need for contraception should be discussed.

Impulsive behaviour is also associated with substance misuse. The risks around substance use and interactions with ADHD medication, including risks for addiction, need to be discussed.

Considerations around pregnancy, the post-partum period and parenting may also be required, since rates of early pregnancy are higher in girls with ADHD. Early pregnancy, may load additional stress and impairment on young girls with ADHD. The consensus group noted difficulties in young ADHD mothers not only in relation to child discipline and behaviour management, but also in relation to the organisational demands of parenting (for example, ensuring bottles are washed, medical and other appointments are kept, child’s clothes are cleaned).

Both individual and group CBT interventions will be helpful in this age-group, the latter providing the opportunity to meet and talk to others who have similar experiences as well as acquire and rehearse social skills in a contained environment.

##### Adulthood

Many of the functional problems experienced by women with ADHD in relation to educational, social, and risk-related behaviours are a continuation of those present in their teenage years. In adulthood, psychoeducation and CBT interventions should continue to address core ADHD symptoms, executive dysfunction, comorbid conditions and dysfunctional strategies (e.g. substance abuse, deliberate self-harm). However, specific attention may be required to address the more complex situations adult females may face, e.g. multitasking occupational demands, home management and family/parenting responsibilities. It is important to encourage the patient to identify and focus on their strengths and positive attributes rather than solely on perceived weaknesses and failures.

Interventions need to address the potential for women with ADHD to be vulnerable in terms of their sexual behaviour and relationships, to support their sexual health and safety. Social stigma associated with risky sexual behaviour in women may augment social problems and limit occupational opportunities. In combination with low self-esteem, this may render women with ADHD vulnerable to sexual harassment, exploitation, and/or abusive or inappropriate relationships. The Adult Psychiatric Morbidity household survey conducted in England found that 27% of females who experienced extensive physical and sexual violence had ADHD traits [189].

The bulk of household, and parental and caring duties are often borne by women [190–192], reflecting social and cultural constraints and expectations. These may result in increased impairment and anxiety in relation to these roles and duties in women compared with men. The consensus group identified that the demands placed on mothers often differ from those of fathers and that low self-esteem may be related to perceived failure to reach societal expectations. Mothers may lack confidence or experience feelings of guilt over their perceived inadequacy as a parent. Dysfunctional beliefs of this nature may be reinforced if they have a difficult-to-manage child with ADHD and are offered ‘parent training’ interventions. The group acknowledged that the term ‘parent training’ is unhelpful and may be perceived as pejorative.

However, at the same time harsh, lax or negative parenting styles have been identified to be elevated in mothers with ADHD [193]. Mothers with ADHD may benefit from life skills coaching, guidance and support in parenting, including ancillary support around parenting strategies. This may be particularly helpful for more vulnerable mothers: those that are young, are sole caregivers for their children, and/or are parenting a child with ADHD. Tailored assessments, support plans and social interventions may help to improve outcomes for this vulnerable group.

Women with ADHD may experience problems in the workplace, such as disorganisation, forgetfulness, inattention, accepting constructive criticism and appraisal, and difficulties managing interpersonal relationships with colleagues. This is likely to be exacerbated in the presence of concurrent intellectual dysfunction and/or other comorbidity. For these types of problems, often a group intervention is helpful and cost-effective. However the decision of whether a group or individualised approach is preferable should be based on careful formulation and individual need. Women may also benefit from targeted support in managing feelings of stress and distress, managing and regulating emotions, coping with rejection and/or feelings of isolation, managing interpersonal conflict, assertiveness training, compromise and negotiation steps, which may help to improve their occupational outcomes and their ability to cope with everyday social interactions.

### Multi-agency liaison

This section addresses issues that arise at a broader institutional level. Primarily, support for females with ADHD may be improved through the psychoeducation and training of individuals who work within these institutions. Some may act as referral gatekeepers and, as such, they have the potential to support or hinder the referral process and to positively or negatively influence the progress of young people and adults within these institutions. A brief summary of multi-agency liaison recommendations is presented in Table 6.

**Table 6:** Multi-agency liaison for ADHD in girls and women: key recommendations

***Educational considerations and adjustments***	
• Training to improve ADHD detection and referral should be provided across teaching and non-teaching educational staff.	
• Students who have or who are suspected of having specific learning difficulties should be screened for ADHD, since young people with ADHD may also present with difficulties in reading and writing.	
• Reasonable adjustments to education provision should be implemented for students with ADHD (e.g. more examination time).	
• Technology Enhanced Learning may support academic and psychosocial education.	
• Proactive planning regarding educational transitions should be made with the student with ADHD, the school and others involved in the student’s care, as appropriate.	
• Flexible learning systems and support with childcare needs may help women with ADHD return to education after having a baby.	
• Career planning should consider non-linear progressions in education and employment, taking into account strengths and weaknesses rather than focusing on current performance.	
***Occupational considerations and adjustments***	
• Women who disclose their disability to their employer are entitled to reasonable adjustments to the workplace in relation to their needs.	
• Additional psychoeducational support may be required to help women manage social and occupational demands in the workplace.	
• Diaries, itineraries, lists, reminder notes and similar scaffolding techniques can be adapted to individual needs through a wide range of digital apps currently available at low or no cost.	
• Returning to or starting work for the first time after children may be a challenge for young women with ADHD.	
***Social care***	
• Training to improve ADHD detection and referral should be provided to staff in all social, family and foster care services.	
• All children at risk of entering the care system should be systematically screened for developmental disorders, including ADHD.	
• Staff should understand that parenting difficulties may be attributed to undiagnosed ADHD rather than a chaotic lifestyle choice, and understand that family members may share symptoms and suffer with associated impairments.	
• Social and family services will benefit from training on psychoeducational input to support young mothers of ADHD children and/or young mothers with ADHD (i.e. to develop skills and coping strategies to help them manage their own mental health and personal needs and those of their child).	
***Criminal justice system***	
• Training to improve ADHD detection and referral should be provided to individuals working in the criminal justice system.	
• Females with ADHD who are in the criminal justice system are unlikely to have a prior diagnosis of ADHD.	
• Full recommendations are provided in a previous consensus meeting [194].	

#### Educational considerations and adjustments

ADHD is associated with low educational attainment and academic underachievement [99, 146, 195]. Interventions should focus on supporting attendance and engagement with education to avoid early school leaving, diminished educational attainment, and associated vulnerabilities. Since ADHD is classified as a disability under the UK Equality Act [196], reasonable adjustments to education provision are mandated (examples may include: additional examination time, academic coaching, rest-breaks during examination, or possibility for part-time study [197]). Research suggests that simple interventions, including physical adjustments (table set-up, creating a time-out corner), and behaviour management techniques, as well as joint goal setting with primary age children, can help to improve ADHD symptoms, social and emotional functioning, and reduce conduct problems in the classroom [198]. However, adjustments cannot be put in place unless ADHD is first recognised and diagnosed.

Young people affected by ADHD are at increased risk for repeating grades, dropping out of high school, being suspended or expelled, and failing to obtain school or higher education qualifications [85, 99, 199]. Maintaining strong links with school ﻿is key to promoting adolescent health and social development [110]. Whilst early or unplanned pregnancy is associated with a reduction in educational and occupational opportunities, school achievement problems in adolescent girls with ADHD have also been shown to predate and predict risky sexual behaviour and unplanned pregnancy [200]. The consensus group noted that exclusion, truancy and school phobia are associated with increased vulnerability of teenage girls with ADHD in relation to later substance misuse, antisocial behaviour, criminality, sexual exploitation and early pregnancy. There is a danger that punitive measures may be harsher for girls who display hyperactive or disruptive symptoms, due to this behaviour constituting a greater violation of social norms and expectations. Excessive punitive measures can lead to loss of engagement with education. Disciplinary problems (e.g. suspensions, verbal or written warnings or expulsions) predict earlier discontinuation of education in boys with ADHD [201], although disciplinary problems are less commonly reported in girls [85].

Externalising conditions have a stronger impact on behaviour in class, whilst internalising problems may impact on motivation and ability to engage in education. Girls with ADHD may present as easily distracted, disorganised, overwhelmed and lacking in effort or motivation. Inattention is more highly predictive of educational under-achievement compared with hyperactivity [202, 203]. Females who are more likely to have the diagnosis missed or misdiagnosed, may be particularly disadvantaged since treatment with ADHD medication has been found to mediate educational outcome. For example, a large-scale study of cross-sectional and longitudinal data in ﻿~10,000 12-year old twins from the Netherlands Twin Register showed the potential efficacy of treatment on academic outcomes [203]. Children taking ADHD medication scored significantly higher on an educational achievement test than children with ADHD who did not.

Individuals with ADHD and intellectual impairments, both male and female, present with complex needs that make it harder for them to engage in education. Many young people with ADHD will have associated specific learning difficulties such as dyslexia, dyscalculia and dysgraphia. Presenting problems may be attributed solely to these specific learning difficulties and/or ASD because school staff are more familiar with them and have a more limited knowledge about ADHD. It may be helpful for students (at all levels of education) who have or who are suspected of having specific learning difficulties to be screened for ADHD, since young people with ADHD may also present with difficulties in reading and writing.

It is important that both child and adult educational professionals have an understanding of ADHD in girls and young women, recognise its presentation and associated vulnerabilities, and have access to screening tools. Training should be disseminated broadly across school staff, including teachers and special educational needs coordinators, as well as teaching assistants, school lunch aides, and after-school club staff who are more likely to supervise children during less structured periods of the day or during one-to-one work in classrooms. It is important that key personnel avoid over-simplistic causation when assessing individual needs (e.g. focusing on their family situation) and understanding of the bi-directional nature of ADHD difficulties in terms of family relationships.

All educational staff should be trained in how to screen females for ADHD and how to make onward referrals for treatment, if indicated. School staff should be trained on the importance of early detection, educational needs and interventions and support strategies that can improve educational outcomes. Training sessions should raise awareness of the current bias towards males in the clinical referral process. Teaching staff may not be as aware of the benefits of referral and ADHD treatment in girls [45], and children with the inattentive subtype [204]. Addressing gender-specific ADHD issues, and gender expectations and stereotypes may help staff to better identify affected females. If ADHD is suspected, schools may consider adopting sensitive screening tools for ADHD (Table 4) or broader mental health problems (e.g. the SDQ [116]). These tend to be cost-effective, quick and reliable, and can help to identify vulnerable girls and young women. Difficulties can arise in maintaining medication treatment programmes in school and staff should be mindful that children may find this stigmatising, especially those who require short-acting medications to be dispensed at school.

Many of the training needs for educational staff remain the same in secondary as in primary school. However, transition to secondary school is accompanied by increased academic demands, and increased requirement for self-organisation and personal responsibility against a backdrop of navigating a new social environment. Young people with ADHD are likely to find this shift in self-management and responsibility especially challenging. ADHD symptoms may become exacerbated and more noticeable, triggering referral for the first time. Good learning and teaching practices (i.e. not necessarily ADHD specific) may help to mitigate many of the potential issues in the classroom by promoting engagement, increasing on-task behaviour and reducing social friction.

Efforts toward Technology Enhanced Learning or e-Learning, are likely to be especially helpful for young people with ADHD. With the appropriate content and support, these learning resources have the potential to go beyond improving academic outcomes in secondary school by improving psychosocial functioning (e.g. helping young people to acquire skills to manage risks of exploitation, bullying and/or victimisation in the school environment or online via social media and communication platforms). Although further research is required to determine the efficacy of e-learning methods for improving outcomes in ADHD, specific examples of successful application of these technologies have been reported (reviewed in [205]).

Careers advice should consider the strengths and weaknesses of female students rather than focus solely on current performance, bearing in mind the relative developmental delay, underachievement, immaturity (and sometimes naivety) of young people with ADHD. Research indicates that occupational ‘fit’ can serve to exacerbate or reduce impairments associated with ADHD. For example, some individuals with ADHD show a preference for more stimulating environments, active, hands-on, or busy and fast-paced jobs [206]. Career planning that incorporates work experience, non-linear progression towards tertiary education and opportunities to re-sit exams or demonstrate potential may be beneficial for those who have struggled to sustain their engagement in a formal school setting.

Guidance for those wishing to embark in further education should take account of the course demands involved (e.g. level of coursework, method of examination). For those who move away from home, transition is further complicated by the many challenges involved in independent living such as financial management, taking responsibility for domestic and occupational arrangements and healthcare. Moving away from home often escalates social demands, with pressure to integrate with people of different ages, cultural backgrounds and interests. It is essential that young people with ADHD make supportive links within the educational organisation (e.g. disability services or student support services) who can support them to access the help to meet their needs, and coordinate with primary health services. This needs to be planned and thought through in advance because a lack of structure and support at this key stage of transition may unveil or amplify ADHD symptoms, together with associated clinical and functional impairments. Adequate support can help young people with ADHD access additional resources. For example, students with ADHD in further or higher education can apply for Disabled Students Allowance (https://www.gov.uk/disabled-students-allowances-dsas), which can fund assistive technology (e.g. speech to text software), specialist mentoring (to help with organisational and planning skills) and “academic coaching”.

In general young people with ADHD reach or complete higher education at a later age than their peers [201]. This can be due to having to repeat years, re-take modules, and obtain extensions for coursework. Many drop out early due to educational or social problems, or early pregnancy. This emphasises the importance for young people having the opportunity to re-access education in later years. However whilst special educational needs support may be available up to age 25 in the UK, women with unrecognised ADHD may experience difficulties in accessing these provisions or meeting eligibility criteria for learning difficulties. Flexible learning systems and support with childcare are helpful initiatives, e.g. in the UK women with children who wish to return to education can obtain childcare support through government initiatives, such as Care to Learn (https://www.gov.uk/care-to-learn), and Childcare Grants (https://www.gov.uk/childcare-grant).

#### Occupational considerations and adjustments

In adulthood, ADHD is associated with unemployment or working in unskilled occupations [201], difficulty maintaining jobs [99, 201], and impaired work performance and financial stress [207]. A longitudinal study following up girls age from eight until age 30, found that women with childhood ADHD were more likely than their peers to have no or few qualifications, be in poorly paid employment, claim benefits, live in temporary or social housing and have a low income [68].

ADHD qualifies as a disability under the UK Equality Act 2010 [196], because it can have a substantial and long-term impact on a person’s ability to perform day-to-day activities. This status can afford women with ADHD certain rights, and access to certain services. For women with ADHD commencing employment, additional support may be required regarding the decision to disclose they have a disability. They may need support in understanding the demands of an organisation, the work-role and personnel structure, how to manage interpersonal conflict, and guidance on how to manage their time, plan and prioritise tasks. Diaries, itineraries, lists, reminder notes and similar scaffolding techniques can be adapted to individual needs through a wide range of digital apps currently available at low or no cost.

Women with ADHD may experience particular difficulty returning to work after having children. This is associated with employment penalties linked to educational problems and potentially having left school early with few or no qualifications. Initiatives such as Specialist Employability Support (https://www.gov.uk/specialist-employability-support) are available to provide intensive support and training for unemployed people with a disability.

Occupational difficulties may be further compounded by a difficulty managing the effects of persisting ADHD symptoms on job-related and social performance in the workplace, together with the need to balance occupational demands with childcare. Reasonable adjustments in the workplace may be helpfully put in place [208] but these may only be achieved if women with ADHD elect to disclose they have a disability. This may not be an easy decision as the individual must balance the need to optimise the environment against their fear of social and occupational stigma, the latter including the possibility they may be held back in promotion and/or other career advancement.

On the other hand, disclosing a disability allows for women with ADHD to be treated more favourably under the UK Equality Act 2010 [196], and benefit from reasonable adjustments that remove barriers in the workplace that would otherwise disadvantage them. Reasonable adjustments are assessed on a case by case basis and extra support for the costs of making reasonable adjustments in the workplace can come from the Access to Work government initiative (see: https://www.gov.uk/access-to-work ). These rights apply to women with ADHD returning to work, taking up employment or becoming diagnosed at any time during their working lives. Employers who fail to comply with this duty would be liable for disability discrimination.

#### Health and social care

Research suggests an increased involvement of ADHD children with the social care and foster care systems [209, 210]. Equipping social care professionals with tools similar to those used in school settings (e.g. the SDQ) may promote a higher level of insight and understanding. Males may be overrepresented in these systems due to high rates of comorbidity with disruptive behavioural problems. Females with ADHD may be more likely to come into contact with social services if they are young single parents struggling with child-care responsibilities; however their underlying ADHD may be unrecognised.

The overrepresentation of developmental disorders in the care population may be the result of a failure in existing services to recognise the specific contribution of these conditions to family breakdown, and an absence of targeted support in such cases. The group recommends that all children at risk of entering the care system should be systematically screened for developmental disorders. Social care professionals may struggle to identify the parenting potential in undiagnosed women with ADHD, and attribute difficulties more to a chaotic lifestyle choice rather than to any underlying disorder. Given the high heritability rates [132] it is also helpful to consider that other family members may also share symptoms and suffer with associated impairments, when examining family dynamics.

Social and family services will benefit from training so they can provide specific psychoeducational input to support young mothers of ADHD children and young mothers with ADHD. If deemed appropriate, they might refer mothers with ADHD to mental health services for targeted support that aims to develop skills and coping strategies, and to help them manage their own mental health and personal needs and those of their child.

The early sexual activity, promiscuity and higher risk for sexually transmitted diseases in some females with ADHD is likely to increase contact with sexual health clinics. ADHD training should therefore be extended to include service-providers at these clinics in order to raise awareness of the presentation and needs of females with ADHD. For example this may lead to better understanding of the need for additional sexual health education, including digital health education, which in turn may better support these young women and prevent sexual exploitation.

#### Criminal justice system

Increased rates of delinquent or criminal behaviour may lead to contact with the criminal justice system [107]. Prevalence of ADHD in incarcerated populations is high, estimated at around one quarter (25.5%) but with no significant differences overall in relation to gender or age. There is however a lower prevalence in adult women than men (22.1% in female adults v. 31.2%, male adults), whereas female youths have a similar prevalence to male youths (30.8% and 29.5%, respectively) [107]. One study reported that only 18.8% of male adult offenders diagnosed with ADHD in prison had a prior diagnosis of ADHD [211]. It is likely that this proportion is even lower for females.

Evidence indicates that ADHD treatment is associated with reduced rates of criminality [212], is tolerated and effective in prison inmates [213], and improves their quality of life and cognitive function [214]. This has led to speculation that effective identification and treatment of ADHD may help to reduce reoffending, albeit with reservations surrounding potential for diversion or misuse of medications, treatment adherence, and discontinuity of ADHD treatment after release [215]. Current best practice recommendations for screening, identifying, treating and supporting ADHD in prisoners and youth offenders are provided in a previous review and consensus report [194], with particular recommendations for support provided for female offenders.

Females with ADHD are likely to be perceived to deviate substantially from stereotypical expectations of behaviour. The differential diagnosis between BPD and ADHD may be particularly important for females in forensic settings, where a high rate of comorbidity has been reported [216]. In the criminal justice system, including prison, there may possibly be a more sympathetic approach toward female offenders but, as for males, their ADHD is unlikely to be recognised. The group noted that ADHD is commonly perceived as ‘bad behaviour’ rather than a vulnerability in this setting, perhaps reflecting high rates of critical incidents (verbal and physical aggression, damage to property, self-injury) being reported in prison [217]. This may be intensified in female offenders with ADHD due to poor understanding of the condition. Further research regarding the interface between the criminal justice system and females with ADHD is needed.

## Discussion

Over 30 years ago, Berry, Shaywitz and Shaywitz warned that girls constitute a ‘silent minority’ in ADHD, with more internalised behaviour making them less likely to be referred for assessment [36]. This does not appear to have changed. Females with ADHD remain more likely to be unrecognised or mis-identified leading to lower than expected rates of referral, assessment and treatment for ADHD. Whilst this has been attributed to the higher rate of internalised and inattentive only presentation in girls, this omission is remarkable, given that the predominantly inattentive subtype of ADHD has been endorsed by the Diagnostic and Statistical Manual, a key diagnostic tool, for many years.

There are specific barriers that seem to hinder the recognition of ADHD in girls and women. These include symptomatic differences, gender biases due to stereotypical expectations, comorbidities and compensatory functions, which mask or overshadow the effects of ADHD symptoms. There is strong public perception that ADHD is a behavioural disorder that primarily affects males. Hence the challenge is to raise awareness and provide training on the presence and presentation of ADHD in females to agencies that regularly interface with children, young people and adults.

The current health and social care system appears to be better geared toward identifying and treating ADHD presenting alongside behavioural and externalising problems, in particular those that present as overt, disruptive and aggressive in nature, and are more commonly seen in boys and men. It is erroneous to consider that females do not present with hyperactive and impulsive symptoms – they do. However, these are generally less overt and aggressive in nature than the conduct problems displayed by males and instead seem to relate to more social-relational and psychosexual problems and behaviours. Understanding the expression of ADHD in females is the first step towards improving detection, assessment, and treatment, and ultimately enhancing long-term outcomes for girls and women with ADHD.

One of the most consistent topics discussed at the consensus (and across all breakout groups) related to how social-relational and psychosexual problems seem to be more marked in females with ADHD compared with males. Difficulties in managing and maintaining functional interpersonal relationships hinder some girls and women from developing or maintaining a positive social network or accessing peer support. ADHD symptoms and emotional lability seem to be related to dysfunctional coping strategies and dissatisfaction with life [77]. Lack of planning for the future [86] may mean that girls and women with ADHD lack constructive activities and occupations in adulthood. These effects may lead to affected girls and women becoming overwhelmed, anxious and low in mood. In turn they may respond by applying dysfunctional coping strategies, such as self-harm and substance use.

Females with ADHD overall have an earlier onset of sexual activity, more sexual partners, and an increased risk of contracting sexually transmitted infections or having an unplanned pregnancy. They are at risk of sexual exploitation, perceived exhibitionism or being considered promiscuous. Social stigma associated with risky sexual behaviour in women may augment social problems, and render affected women vulnerable to being victimised, bullied, harassed, abused, or entering into unhealthy relationships. Young girls with ADHD may become young mothers with ADHD (and possibly also mothers of children with ADHD). This is associated with a further reduction in educational and occupational opportunities. Research is needed to tease out the motivations and causal mechanisms of these behaviours and outcomes in females with ADHD, and if, how and why they may differ from those of males.

Treatment has been reported to moderate the lifetime risks of ADHD for both males and females. The consensus group identified where adjustments to approaches in treatment are needed to better support girls and women with ADHD. This includes more frequent treatment monitoring and psychoeducation at times of personal transition, with a greater focus on functional and emotional aspects of the disorder. The consensus group considered that multi-agency liaison will also be needed to support some girls and women with ADHD. Furthermore, raising awareness of, and providing training about, ADHD in institutions (e.g. educational, social, family, sexual health and criminal justice services) as well as the key healthcare system (primary health, child and adolescent mental health services and adult general psychiatry) will be helpful to improve detection of girls and women with ADHD, increase understanding and reduce stigma.

The consensus highlighted the relative dearth of research on the life-span experience of females with ADHD. Given the higher prevalence of ADHD in males, it would be helpful if studies reporting sex-mixed cohorts segregated data and results by gender. This would be particularly helpful in large clinical or population-based studies, where information on girls with ADHD would otherwise be buried as variance under the predominant male group. Providing sex-segregated results and data for all studies of ADHD (perhaps under supplementary data) would provide information to inform future meta-analyses.

Future research should investigate the presentation and needs of females with ADHD: how they might better be identified and assessed, and how their treatment response should best be evaluated and monitored to effectively improve outcomes. The most recent meta-analyses of gender differences in ADHD symptom presentation and associated features was reported over 15 years ago. An updated meta-analysis including all recent data is now needed. More research is also required to elucidate the interaction of hormones, ADHD symptoms and stimulant medication on functioning during key times of hormonal change (e.g. during the menstrual cycle, pregnancy and the postpartum period, and menopause), to help inform treatment plans. Factors that are associated with hyperactive/impulsive symptoms in females with ADHD and how these differ to males should be investigated further, including sexual behaviours and their motivations in girls and women with ADHD, as well as vulnerabilities to victimisation, physical and sexual assault and cyberbullying.

## Conclusions

This consensus will inform effective identification, treatment and support of girls and women with ADHD. To facilitate identification, it is important to move away from the previously predominating ‘disruptive boy’ stereotype of ADHD and understand the more subtle and internalised presentation that predominates in girls and women. In treatment, it is important to consider a lifespan model of care for females with ADHD, which supports the complex and developmentally changing presentation of ADHD in females. Appropriate intervention is expected to have a positive impact on affected girls and women with ADHD, their families, and more broadly on society leading to increased productivity, decreased resource utilization and, most importantly, better outcomes for girls and women.

## Data Availability

Data sharing is not applicable to this article as no datasets were generated or analysed during the current study.

## Abbreviations

ACE: ADHD Child Evaluation
ADHD: Attention-Deficit/Hyperactivity Disorder
ASD: Autism Spectrum Disorder
ASRS: Adult ADHD Self-report Rating Scale
BPD: Borderline Personality Disorder
CAARS: Conners’ Adult Rating Scales
CBT: Cognitive Behavioural Therapy
CBRS: Conners’ Comprehensive Behavior Rating Scales
CD: Conduct Disorder
CPT 3: Conners’ Continuous Performance Test, third edition
DAWBA: Development and Wellbeing Assessment
DIVA: Diagnostic Interview of Adult ADHD
DIVA-5 ID: Diagnostic Interview for ADHD in Adults with Intellectual Disability
DSM: Diagnostic and Statistical Manual of Mental Disorders
EHCP: Education, Health and Care Plan
ICD: International Classification of Diseases
K-SADS-PL: Schedule for Affective Disorders and Schizophrenia for School-Age Children-Present and Lifetime Version
LD: Learning Disability
ODD: Oppositional Defiant Disorder
PEP: Personalised Education Plan
VARS: The Vanderbilt ADHD Rating Scales
QbTest: Quantified Behavior Test
SDQ: Strengths and Difficulties Questionnaire
SNAP: Swanson, Nolan, and Pelham-IV Questionnaire
SUD: Substance Use Disorder
UK: United Kingdom of Great Britain and Northern Ireland
UKAP: United Kingdom ADHD Partnership
